# Dynamic ^15^N{^1^H} NOE measurements: a tool for studying protein dynamics

**DOI:** 10.1007/s10858-020-00346-6

**Published:** 2020-09-12

**Authors:** Vladlena Kharchenko, Michal Nowakowski, Mariusz Jaremko, Andrzej Ejchart, Łukasz Jaremko

**Affiliations:** 1 Division of Biological and Environmental Sciences and Engineering (BESE), King Abdullah University of Science and Technology (KAUST), Thuwal, 23955-6900 Saudi Arabia; 2 Faculty of Chemistry, Biological and Chemical Research Centre, University of Warsaw, Żwirki i Wigury 101, 02-089 Warsaw, Poland; 3 Institute of Biochemistry and Biophysics, Polish Academy of Sciences, Pawinskiego 5A, 02-106, Warsaw, Poland

**Keywords:** NMR, Protein dynamics, Heteronuclear NOE, Dynamic NOE, Errors of NOE measurements

## Abstract

Intramolecular motions in proteins are one of the important factors that determine their biological activity and interactions with molecules of biological importance. Magnetic relaxation of ^15^N amide nuclei allows one to monitor motions of protein backbone over a wide range of time scales. ^15^N{^1^H} nuclear Overhauser effect is essential for the identification of fast backbone motions in proteins. Therefore, exact measurements of NOE values and their accuracies are critical for determining the picosecond time scale of protein backbone. Measurement of dynamic NOE allows for the determination of NOE values and their probable errors defined by any sound criterion of nonlinear regression methods. The dynamic NOE measurements can be readily applied for non-deuterated or deuterated proteins in both HSQC and TROSY-type experiments. Comparison of the dynamic NOE method with commonly implied steady-state NOE is presented in measurements performed at three magnetic field strengths. It is also shown that improperly set NOE measurement cannot be restored with correction factors reported in the literature.

**Electronic supplementary material:**

The online version of this article (doi:10.1007/s10858-020-00346-6) contains supplementary material, which is available to authorized users.

## Introduction

Since its first use of magnetic relaxation measurements of ^15^N nuclei applied to the protein, the staphylococcal nuclease (Kay et al. 1989), this method has become indispensable in the determination of molecular motions in biopolymers (Jarymowycz and Stone 2006; Kempf and Loria 2003; Palmer, III 2004; Reddy and Rayney 2010; Stetz et al. 2019). The canonical triad of relaxation parameters—longitudinal (*R*_1_) and transverse (*R*_2_) relaxation rates accompanied by the ^15^N{^1^H} nuclear Overhauser effect (NOE)—have been most often used in studies investigating the mobility of backbone in proteins. It is a common opinion that ^15^N{^1^H} NOE is unique among the mentioned three relaxation parameters because it is regarded as essential for the accurate estimation of the spectral density function at high frequencies (ω_H_ ± ω_N_), and it is crucial for the identification of fast backbone motions. (Idiyatullin et al. 2001; Gong and Ishima 2007; Ferrage et al. 2009).

The most common method for the determination of X{^1^H} NOE is a steady-state approach. It requires measurements of the longitudinal polarization at the thermal equilibrium of spin X system, *S*_0_, and the steady-state longitudinal X polarization under ^1^H irradiation, *S*_*sat*_ (Noggle and Schirmer 1971). Note that the nuclear Overhauser *effect*, defined as $\varepsilon = {{S_{sat} } \mathord{\left/ {\vphantom {{S_{sat} } {S_{0} }}} \right. \kern-\nulldelimiterspace} {S_{0} }}$, should not be mistaken with nuclear Overhauser *enhancement*, $\eta = {{\left( {S_{sat} - S_{0} } \right)} \mathord{\left/ {\vphantom {{\left( {S_{sat} - S_{0} } \right)} {S_{0} = \varepsilon - 1}}} \right. \kern-\nulldelimiterspace} {S_{0} = \varepsilon - 1}}$ (Harris et al. 1997).

It has to be pointed out that NOE measurements appear to be very demanding and artifact prone observations. One of severe obstacles in these experiments is their *ca*. tenfold lower sensitivity in comparison to *R*_1*N*_ and *R*_2*N*_ which is due to the fact that the NOE experiments with ^1^H detection start with the equilibrium ^15^N magnetization rather than ^1^H. The steady-state ^15^N{^1^H} NOEs (ssNOE) are normally determined as a ratio of cross-peak intensities in two experiments—with and without saturation of H_N_ resonances. Such arrangement creates problems with computing statistically validated assessment of experimental errors. ^15^N{^1^H} NOE pulse sequence requires a very careful design as well. Properly chosen recycle delays between subsequent scans and saturation time of H_N_ protons have to take into account the time needed to reach the equilibrium or stationary values of ^15^ N and H_N_ magnetizations (Harris and Newman 1976; Canet 1976; Renner et al. 2002). Exchange of H_N_ protons with the bulk water combined with the long longitudinal relaxation time of water protons leads to prolonged recycle delay in the spectrum acquired without saturation of H_N_ resonances. Unintentional irradiation of the water resonance suppresses H_N_ and other exchangeable signals owing to the saturation transfer and many non-exchangeable ^1^H resonances via direct or indirect NOE with water (Grzesiek and Bax 1993) while interference of DD/CSA relaxation mechanisms of ^15^N amide nuclei disturbs the steady-state ^15^N polarization during ^1^H irradiation (Ferrage et al. 2009). All aforementioned processes depend directly or indirectly on the longitudinal relaxation rates of amide ^1^H and ^15^N nuclei *R*_1*H*_ and *R*_1*N*_ as well as the longitudinal relaxation rate of water protons, *R*_1*W*_, and the exchange rate between water and amide protons, *k*.

In this study, the dynamic NMR experiment (DNOE), a forgotten method of the NOE determination in proteins, was experimentally tested, and the results were compared with independently performed steady-state NOE measurements at several magnetic fields for widely studied, small, globular protein ubiquitin. Additionally, several difficulties inherent in ^15^N{^1^H} NOEs and methods for overcoming or minimizing these difficulties are cautiously discussed.

## Experimental

The uniformly labeled U-[^15^N] human ubiquitin was obtained from Cambridge Isotope Laboratories, Inc in lyophilized powder form and dissolved to 0.8 mM protein concentration in buffer containing 10 mM sodium phosphate at pH 6.6 and 0.01% (*m*/*v*) NaN_3_. DSS-*d*_6_ of 0.1% (*m*/*v*) in 99.9% D_2_O was placed in a sealed capillary inserted into the 5 mm NMR tube.

Amide resonance assignments of ubiquitin were taken from BioMagResBank (BMRB) using the accession code 6457 (Cornilescu et al. 1998).

NMR experiments were performed on three Bruker Avance NEO spectrometers operating at ^1^H frequencies of 700, 800 and 950 MHz equipped with cryogenic TCI probes. The temperature was controlled before and after each measurement with an ethylene glycol reference sample (Rainford et al. 1979) and was set to 25 °C. The temperature was stable with maximum detected deviation of ± 0.3 °C. Chemical shifts in the^1^H NMR spectra were reported with respect to external DSS-*d*_6_ while chemical shifts of the ^15^N signals were referenced indirectly using frequency ratio of 0.101329118 (Wishart et al. 1995). The spectral widths were set to 12 ppm and 22 ppm for ^1^H and ^15^N, respectively. The number of complex data points collected for ^1^H and ^15^N dimensions 2048 and 200, respectively. In each experiment, 8 scans were accumulated per FID. Double zero filling and a 90°-shifted squared sine-bell filter were applied prior to Fourier transformation. Data were processed using the program nmrPipe (Delaglio et al. 1995) and analyzed with the program SPARKY (Goddard and Kneller). Resonance intensities were used in calculating relaxation times and NOE values obtained from a nonlinear least-squares analysis performed using Fortran routines written in-house, based on the Newton–Raphson algorithm (Press et al. 2007).

The pulse programs used in this work were based on the HSQC-type *R*_1_(^15^N) and ^15^N{^1^H} NOE experiments (Lakomek et al. 2012). The carrier frequency during ^1^H saturation with 22 ms spaced 180° hard pulses on ^1^H was moved from water frequency to the centre of amide region (8.5 ppm). Evolution times in *R*_1_(^15^N) and dynamic NOE experiments were collected in random order. Reproducibility of experiments was excellent. Therefore, the interleaved mode was not used since it could introduce instabilities of water magnetization (Renner et al. 2002). The list of delays applied in the experiments used in this work is given in Table S3.

## Results and discussion

### Dynamic NOE measurement—introduction

It can be concluded from the Solomon equations (Solomon 1955) that in the heteronuclear spin system X–H, the heteronuclear Overhauser effect is built up with the rate *R*_1_(X) under the condition of proton saturation as shown for the ^13^C-^1^H spin system (Kuhlmann et al. 1970; Kuhlmann and Grant 1971). As a consequence of this observation a dynamic NOE was employed for the simultaneous determination of *R*_1_(^13^C) and ^13^C{^1^H} NOE using Eq. (1)

1 $$S(t) = S_{0} [\varepsilon + (1 - \varepsilon )\exp ( - R_{1} t)]$$

Measurements of time dependent changes of signal intensities *S*(*t*) allow for the determination of *ε*, *R*_1_, and their probable errors, as defined by any standard criterion of nonlinear regression methods. The DNOE can be especially beneficial in studying nuclei with negative magnetogyric ratios since in unfavorable circumstances, nulling of the resonance in a proton saturated spectrum can occur. Therefore, the DNOE has been successfully used in relaxation studies of ^29^Si (Kimber and Harris 1974; Ejchart et al. 1992) and ^15^N (Levy et al. 1976) nuclei in organic molecules. The ^15^N-DNOE has been also investigated in small protein (Zhukov and Ejchart 1999). This approach can be especially profitable in studies of medium to large size proteins displaying highly dynamic fragments.

### Time schedule of NOE measurement

**Fig. 1:**
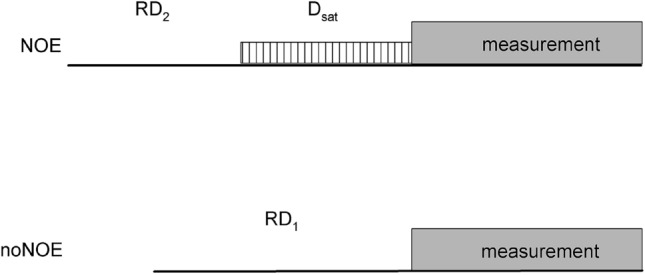
Steady-state NOE measurement is composed of two sequences: NOE and noNOE with saturated and unperturbed H_N_ protons, respectively. Dynamic NOE measurement comprises several NOE type sequences with a set of different *D*_*sat*_ values

Both nitrogen polarizations, *S*_*sat*_ and *S*_0_, depend on a number of physical processes in the vicinity of amide nitrogen nuclei. Dipolar interaction between ^15^N and ^1^H_N_ brings about the nuclear Overhauser effect. Additional processes as chemical shift anisotropy relaxation mechanism of ^15^N and its interference with ^15^N/^1^H_N_ dipolar interaction, direct NOE and saturation transfer from water to ^1^H_N_ protons due to chemical exchange influence both nitrogen polarizations, especially if the pulse sequence itself will result in non equilibrium state of water protons. Presaturation of the water resonance resulting in partial saturation of water magnetization attenuates ^1^H_N_ signal intensities mostly through the chemical exchange or through homonuclear NOE with water protons. (Grzesiek and Bax 1993; Lakomek et al. 2012). Therefore, evolution of the spin system towards *S*_*sat*_ or *S*_0_ nitrogen polarizations depends on the rates of the processes mentioned above, the longitudinal relaxation rates of ^15^N, ^1^H_N_, and water protons, *R*_1*N*_, *R*_1*H*_, and *R*_1*W*_, and the chemical exchange rate, *k*, between amide and water protons. These rates strongly determine the time schedule of NOE measurements, which is schematically shown in Fig. 1. Hence, their knowledge is a prerequisite for choice of optimal delays. The numerical data of *R*_1*H*_ and *R*_1*W*_ for the sample studied here are given in Table 1. Nevertheless, one should be aware that the *R*_1*W*_ depends on temperature, pH, and protein concentration. Residue specific *R*_1*N*_ values for the ubiquitin sample will be discussed further.

**Table 1:** Longitudinal relaxation rates of water protons *R*_1*W*_ and averaged rates of amide protons *R*_1*H*_ for ubiquitin sample at 25 °C

*B*_0_ [T]	*R*_1*W*_ [s^−1^]	*dR*_1*W*_ [s^−1^]	*R*_1*H*_ [s^−1^]	*dR*_1*H*_ [s^−1^]
16.4	0.389	0.003	0.92	0.06
18.8	0.404	0.003	Not determined	
22.3	0.412	0.005	0.85	0.07

Detailed information on the sample composition are given in the experimental results

In the noNOE *reference* measurement, ^15^N nuclei have to reach the thermal equilibrium at the end of delay *RD*_1_. During the block denoted as *measurement* in Fig. 1, the pulse sequence resulting in the 2D ^15^N/^1^H spectrum with the desired cross peak intensities is executed. At the start of acquisition, several coupled relaxation processes take place, resulting in multi-exponential decay of ^15^N, ^1^H_N_, and water protons (Ferrage et al. 2008). Keeping in mind that *R*_1*W*_ is much smaller than the rates of other processes, it can be reasonably assumed that *R*_1*W*_ rate mainly defines *RD*_1_. Fulfillment of the condition

2 $$\exp ( - RD_{1} \cdot R_{1W} ) < 0.02$$

where factor 0.02 has been chosen to some extent arbitrarily, should properly determine *RD*_1_ values in most of the cases. Still one has to be aware that the smallest decay rate resulting from the exact solution of full relaxation matrix can be smaller than *R*_1*W*_.

In NOE measurement, the buildup of ^15^N magnetization takes place with the rate *R*_1*N*_. ^15^N relaxation rates can be, however, broadly dispersed if mobility of N–H vectors in a studied molecule differ significantly. Therefore, to meet the condition

3 $$\exp ( - D_{sat} \cdot R_{1N} ) < 0.02$$

a compromise may be required (c.f. Table S1). Experiments of steady-state and dynamic NOE measurements differ in the *RD*_2_ setting. In the case of steady-state NOE, the value *RD*_2_ = 0 is adequate. Even if the nitrogen polarization displays a nonzero value at the beginning of the *D*_*sat*_ period, it will still have enough time to reach the steady-state condition. In dynamic NOE, however, the nitrogen polarization has to start from closely controlled thermal equilibrium. Therefore, condition (2) with *RD*_1_ replaced with *RD*_2_ has to be fulfilled. The description (*RD*_1_–*RD*_2_–*D*_*sat*_)/*B*_0_ will be further adopted to characterize particular NOE experiments used in this work.

Analysis of systematic errors resulting from an incorrect delay setting in NOE values, ε = *S*_*sat*_/*S*_0_, for nuclei with γ < 0 should take into account that these errors can be caused by false *S*_0_ values and/or *S*_*sat*_ values. The apparent *S*_0,*app*_ value in not fully relaxed spectrum is always smaller than the *S*_0_ of true equilibrium value. On the other hand, the non-equilibrium apparent *S*_*sat,app*_ value is always larger than the *S*_*sat*_, equilibrium value, *i.e.* more positive for ε > 0 or less negative for ε < 0. The joint effect of erroneous *S*_*sat*_ and *S*_0_, however, does not always result in the relation ε_*app*_ > ε as could be hastily concluded. An attenuated *S*_0_ value in conjunction with properly determined, negative *S*_*sat*_ results in ε_*app*_ < ε, and this is experimentally confirmed by ε values observed for the C-terminal, mobile residue G76. Its values obtained in the measurements free of systematic errors (10-10-8)/16.4 T and (10-10-5)/22.3 T are equal to − 0.812 and − 0.246, respectively. Herein, both, *S*_0_ and *S*_*sat*_ values are expected to be error free. In the measurements (10-10-4)/16.4 T and (10-10-1.3)/22.3 T with proper *S*_0_ value and *S*_*sat,app*_ > *S*_*sat*_ owing to too short *D*_*sat*_, ε_*app*_ are equal to − 0.738 and 0.162, respectively, while in (3-0-3)/22.3 T with too short *RD*_1_ and *D*_*sat*_ delays, *S*_0,*app*_ < *S*_0_ and ε_*app*_ =  − 0.379 (cf. Figure 8). Such misleading behavior could be expected for mobile residues in flexible loops, unstructured termini, or intrinsically disordered proteins.

### Setup and data processing of DNOE measurement

**Fig. 2:**
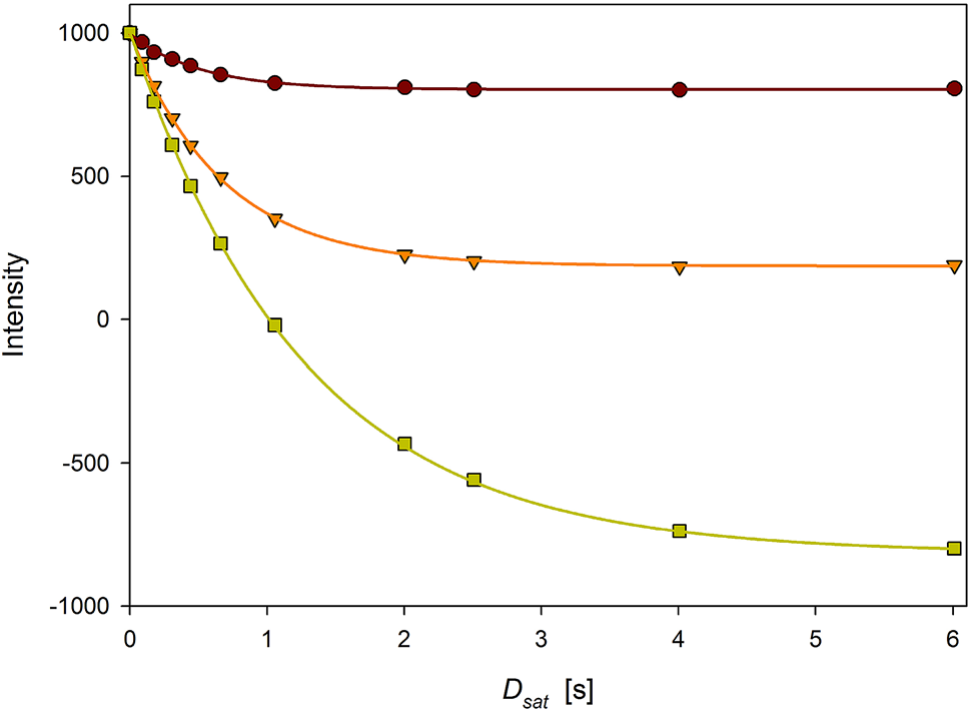
Experimental data obtained in DNOE measurement at 16.4 T for D58 residue (brown circles), R74 (orange triangles), and G76 (light green squares). NOE values determined in the sequential task are: (D58) = 0.805, *ε* (R74) = 0.186, and *ε* (G76) =  − 0.813. Color-coded lines correspond to the nonlinear least-square fit of the Eq. (1) to the experimental data. Correlations between *ε* and *R*_1_, *c*(*ε*,*R*_1_), in the simultaneous task are: *c*(D58) =  − 0.003, *c*(R74) = 0.013, and *c*(G76) = 0.099. The larger range of intensities results in larger correlation *c*(*ε*,*R*_1_) between fitted parameters

Relation between signal intensities and evolution times in a dynamic NOE experiment, *D*_*sat*_, depend on three parameters: nuclear Overhauser effect, *ε*, nitrogen longitudinal relaxation rate, *R*_1*N*_, and signal intensity at the thermal equilibrium, *S*_0_ (Eq. 1). Provided that the longitudinal relaxation rates have been previously obtained in a separate experiment, their values can be entered in Eq. 1, reducing the number of determined parameters in a computational task further denoted as a sequential one. Influence of the propagation of *R*_1*N*_ errors on the *ε* values is usually negligible; variation of *R*_1*N*_ values within the range ± σ (standard deviation) typically results in *dε* changes smaller than 10^–5^ except for residues exhibiting *ε* < 0.4 (Figs. S1, S2). In ubiquitin, such residues are located at the C-terminus. This behavior is attributed to the stronger correlation between *ε* and *R*_1*N*_ parameters owing to the increased range of signal intensities for smaller *ε* values (Fig. 2). Another possibility of data processing, simultaneous use of dynamic NOE and relaxation rate data in one computational task, brings about results (*ε* and *dε* values) practically identical to those obtained in the sequential task.

The dynamic NOE data can also be used without support from separate *R*_1*N*_ data. Such data processing delivers the *ε* values and their errors close to those resulted from the sequential or simultaneous approach (Figs. S3, S5). On the other hand, derived *R*_1_ relaxation rates are less accurate with errors an order of magnitude larger than those obtained in the dedicated *R*_1_ experiment (Figs. S4, S6). Therefore, a dynamic NOE measurement cannot be regarded as a complete equivalence of a separate *R*_1_ experiment. Numerical data for three different data processing methods of dynamic NOE at 22.3 T are given in the Table S2, and a comparison of the discussed numerical methods are presented in Table 2 using data acquired for ubiquitin at 16.4 and 22.3 T. The pairwise root-mean-square deviations (RMSD) for *ε* values are extremely small in all cases, while those for *R*_1_ values are larger. Their values, together with average standard deviations, are given in Table 3. Recently, an experimentally demanding TROSY-based pulse sequence dedicated to deuterated proteins has been invented for simultaneous measurement of *R*_1N_ relaxation rates and *ε* values. The accuracy of the proposed technique has been verified by comparison to the results of both relaxation parameters measured conventionally (O'Brien and Palmer III 2018).

**Table 2:** Values of standard error ratios averaged over 70 amino acid residues of ubiquitin available from our experiments

*B*_0_ [T]	*dR*_1_/*R*_1_ [s^−1^]		dε/ε	
	B/A	C/A	B/A	C/A
16.4	1.11	7.52	1.02	0.99
22.3	0.97	7.59	1.30	0.98

Comparison of the data reduction methods in dynamic NOE experiments: (A) sequential determination of *R*_1_ from the dedicated *R*_1_ measurement followed by the ε determination from DNOE measurement using previously determined *R*_1_ values, (B) simultaneous use of DNOE and *R*_1_ measurements in a single computational task, (C) DNOE measurement data alone used for the determination of *R*_1_ and ε values

**Table 3:** The pairwise RMSDs and the mean values of standard deviations determined for three data reduction methods for dynamic NOE experiments

Pairwise RMSD			The mean of standard deviations		
*B*_0_ = 22.3 T	ε	*R*_1_ [s^−1^]		σ (ε)	σ (*R*_1_) [s^−1^]
A/B	0.0002	0.0082	A	0.0029	0.0081
A/C	0.0016	0.0862	B	0.0036	0.0077
B/C	0.0017	0.0852	C	0.0028	0.0611

Labels A, B, and C are defined in the caption to Table 2

**Fig. 3:**
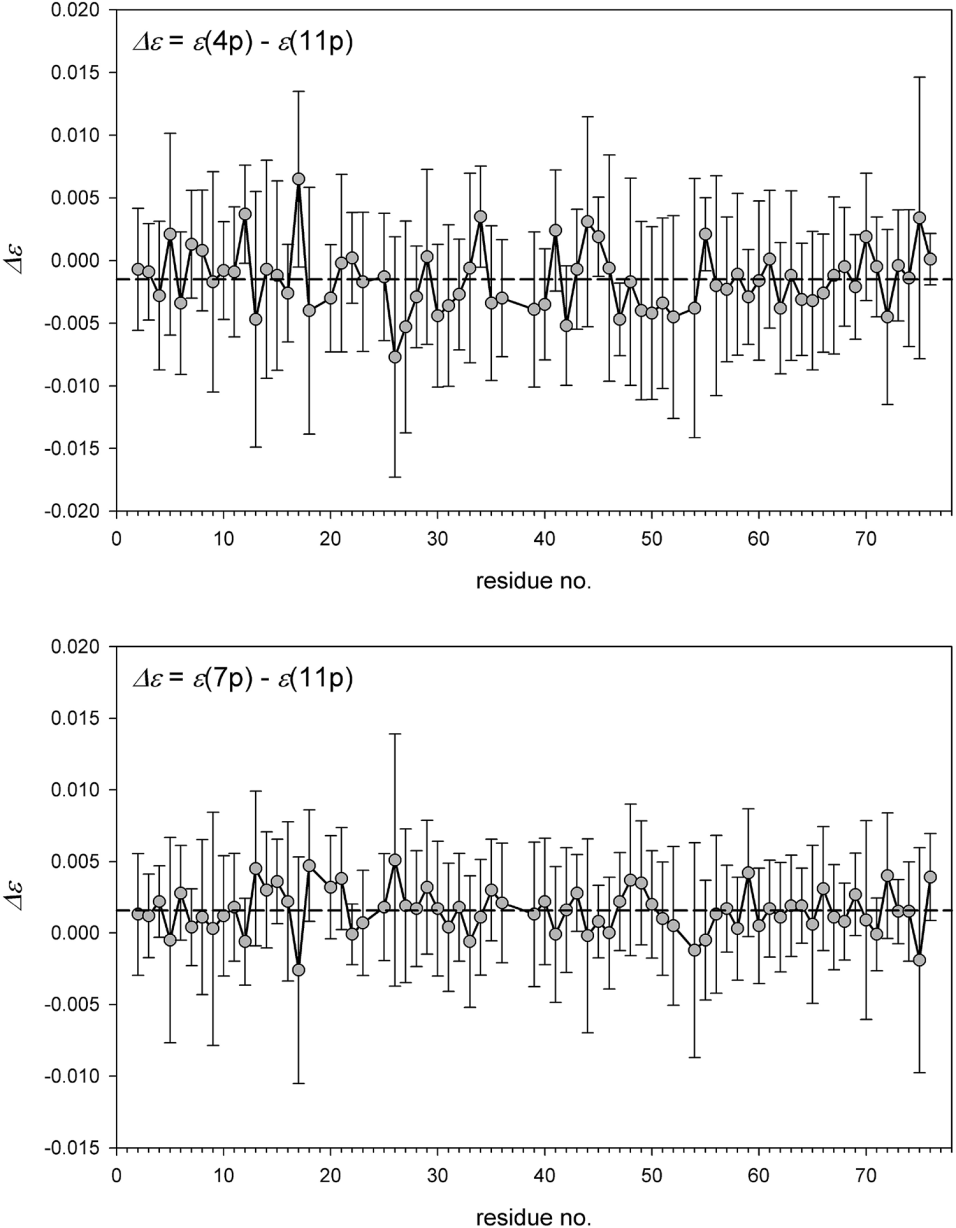
Residue specific differences with error bars between DNOE measurement at 22.3 T comprising 11 *D*_*sat*_ values and curtailed DNOE measurements composed of four or seven *D*_*sat*_ values (upper part and lower part, respectively). Horizontal, dashed lines represent averages of Δ*ε* values given in plots. Full set of *D*_*sat*_ values [0.0, 0.11, 0.22, 0.35, 0.55, 0.66, 0.79, 1.10, 1.30, 3.00, 4.00]. Four values: 0.22, 0.66, 1.10, and 3.00 were rejected to get seven *D*_*sat*_ value measurement. Further rejection of 0.11, 0.55, and 1.30 *D*_*sat*_ values resulted in four-value set

Dynamic NOE measurements, as with relaxation rate experiments, require optimization of a number and length of saturation periods, *D*_*sat*_. One important assumption in the selection of *D*_*sat*_ values is to sample a broad range of intensities *I*(*t*) ~ *S*(*t*) in a uniform manner. The shortest *D*_*sat*_ equal to zero delivers *I*_0_ ~ *S*_0_. The longest *D*_*sat*_ should be as close to a value fulfilling the condition (2) as is practically feasible (c.f. Table S1). These assumptions were checked on the DNOE measurement comprising 11 delays. Next the number of delays was reduced to seven and then to 4 selected delays, and results were compared. Apparent NOE values and their standard deviations changed only slightly. Residue specific differences in *ε* values between the full experiment and each of the reduced ones were smaller than appropriate *dε* values. They are compared in Fig. 3, and the presented data assure that four correctly chosen *D*_*sat*_ values do not deteriorate *ε* values and their accuracies. This conclusion allows us to state that DNOE measurement can require an acceptable amount of spectrometer time.

### Error determination of NOE measurements

The NOE errors are equally important to NOE values themselves. They are used to weigh the NOE data in the relaxation-based backbone protein dynamics calculation (Palmer et al. 1991; d’Auvergne 2008; Jaremko et al. 2015). Inaccurate values of NOE errors can result in the erroneous estimation of protein backbone dynamics. Particularly, the overestimation of NOE leads to significant errors in the local dynamics parameters as evidenced by appropriate simulations (Ferrage et al. 2008). Occasionally, the average values of the NOE and standard errors in the mean have been determined from several separate NOE data sets (Stone et al. 1992; Renner et al. 2002). Nonetheless, it has been most often accepted to use signal-to-noise ratios (*SNR*) in the determination of steady-state NOE errors (Farrow et al. 1994; Tjandra et al. 1995; Fushman 2003).

4 $$d\varepsilon = \left| \varepsilon \right|\sqrt {SNR_{sat}^{ - 2} + SNR_{nonsat}^{ - 2} }$$

The Eq. (4) is an approximation of exact formulation of experimental error determination since it takes into account only this part of experimental errors which arises from the thermal noise. It can be safely used if the thermal noise dominates other contributions to the total experimental error. A weak point in Eq. (4) arises also from the fact that amino acid residues located in flexible parts of macromolecules often display NOE values close to zero, which results in the underestimation of *dε*, owing to the factor $\left| \varepsilon \right|$ as shown in Eq. (4).

**Fig. 4:**
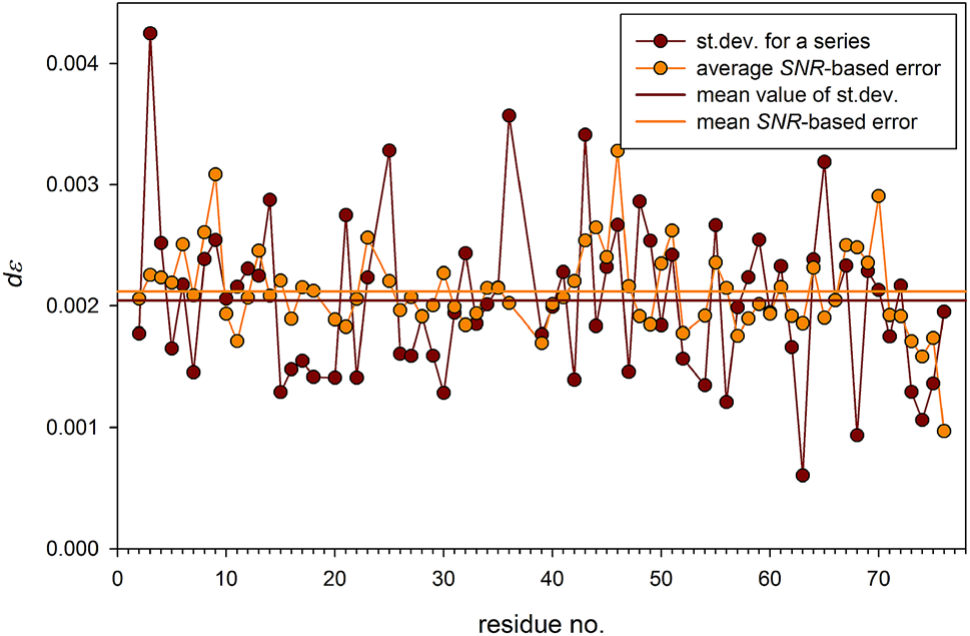
Standard deviations (σ) calculated for 70 residues of ubiquitin (brown circles) and their mean (solid horizontal brown line) determined for series of ten measurements. Means of ten *SNR*-based errors calculated for each residue (orange circles) and their mean (solid horizontal orange line)

**Fig. 5:**
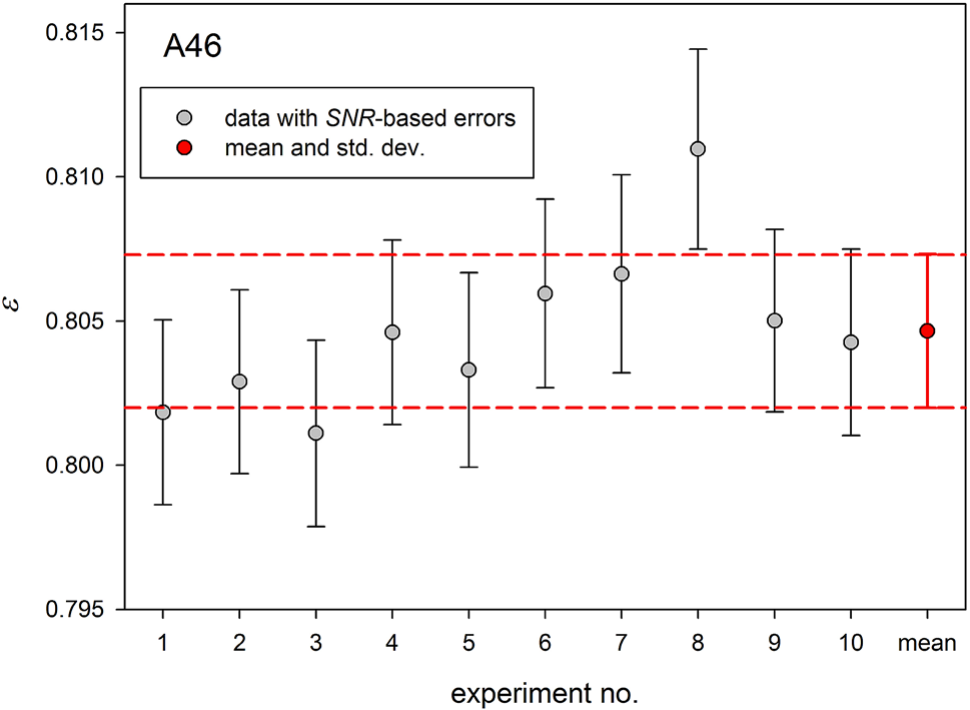
The NOE values of A46 residue obtained in a series of 10 measurements with appropriate *SNR*-based errors (gray circles with *SNR*-based error bars) and their mean with standard deviation (red circle). Dashed red lines correspond to the mean ± σ

Justification of an *SNR*-based approach should comprise two issues: checking of the reliability of *SNR* determination delivered by commonly used processing tools and comparison of the *SNR*-determined errors with those obtained from the statistical analysis of a series of independent NOE measurements. To the best of authors' knowledge, such study has not been yet undertaken for ^15^N nuclei in proteins and has only be performed once for ^13^C nuclei (Bernatowicz et al. 2010). In our study, we found that *SNR* values automatically derived in the peak intensity determination differed from those obtained semi-manually; their larger part was overestimated. Therefore, automatically delivered *SNR* values concomitant cross peak intensities cannot be taken for granted. Description of the *SNR* issue is given in the Supporting Material (section: Determination of signal-to-noise ratio). In order to closely analyze the relevance of *SNR*-based NOE errors, a series of 10 NOE measurements was performed at 22.3 T using identical spectrometer setup. A comparison of standard deviations (*σ*) calculated for each of 70 residues of ubiquitin with corresponding means of *SNR*-based NOE errors is presented in Fig. 4. It can be concluded from Fig. 4 that values of two presented sets of NOE errors are very similar, and their means are close to one another with a difference of 8⋅10^−5^. Individual ε values for the residue A46 showing the largest NOE data dispersion are compared with the mean and the standard deviation in Fig. 5. Examination of Figs. 4 and 5 allows us to conclude that properly determined *SNR*-based NOE errors are reliable and can be safely used in further applications.

### Saturation of H_N_ protons

Originally, saturation of proton resonances was achieved by a train of 250° pulses at 10 ms intervals (Markley et al. 1971). In protein relaxation studies, however, a train of 120° pulses spaced 20 ms apart was commonly used for this purpose (Kay et al. 1989). In search of the optimal ^1^H saturation scheme, different pulse lengths (120°, 180°, 250°) and different pulse spacings (5 ms, 10 ms, 20 ms) were employed (Renner et al. 2002). Finally, it was concluded that pulses of approximately 180° at l0 ms intervals performed slightly better than other settings.

Extensive experimental survey of H_N_ proton saturation accompanied by theoretical calculations based on averaged Liouvillian theory was carried out on all components of saturation sequence (Ferrage et al. 2009, 2010). It was concluded that the best results were obtained using the symmetric 180° pulse train (*τ*/2 − 180° − *τ*/2)_*n*_ with *τ* = *k*/*J*_*NH*_, where *n*—the integer determining length of saturation time (*D*_*sat*_ = *n*⋅ *τ*) and *k*—a small integer, usually *k* = 2, giving *τ* about 22 ms. It was also suggested to move the proton carrier frequency from water resonance to the center of the amide region and reduce the power of the 180° pulses to minimize sample heating.

### Analysis of NOE experiments

NOE experiments performed to analyze the influence of a particular sequence of parameters on the apparent nuclear Overhauser effects values, *ε*_*app*_, are presented in Table S3. Experiments, ssNOE(10-10-8)/16.4, DNOE/16.4, ssNOE(14-0-14)/18.8, ssNOE(13-0-3)/22.3, and DNOE/22.3 can be expected to deliver the most accurate results. They are regarded as a kind of reference point for a selected magnetic field.

**Fig. 6:**
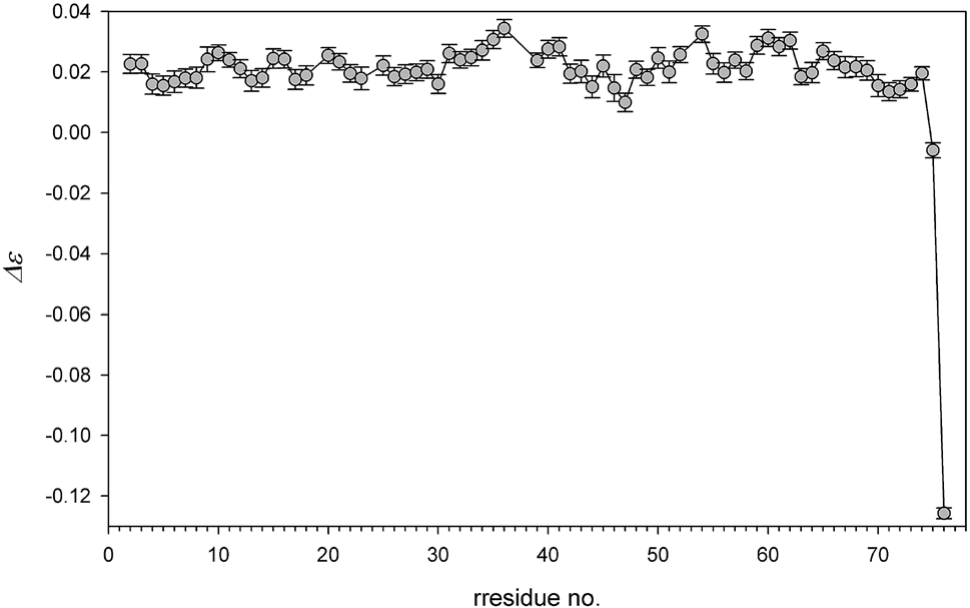
NOE differences Δε = ε_*app*_ − ε obtained in measurements performed at 18.8 T with *D*_*sat*_ = 4 s (ε_*app*_) and *D*_*sat*_ = 14 s (ε). Average difference after rejection of G75 and G76 with ε < 0 is equal to 0.022

The importance of using appropriate *D*_*sat*_ values in steady-state NOE measurements is demonstrated by comparing NOEs in the experiments (14-0-4)/18.8 and (14-0-14)/18.8. The first displays a systematic increase of *ε*_*app*_ owing to incomplete H_N_ saturation during *D*_*sat*_. Residue specific differences between the mentioned experiments are shown in Fig. 6. Residues G75 and G76 with negative *ε* values display decreased *ε*_*app*_ as discussed earlier (section: Time schedule of NOE measurement).

**Fig. 7:**
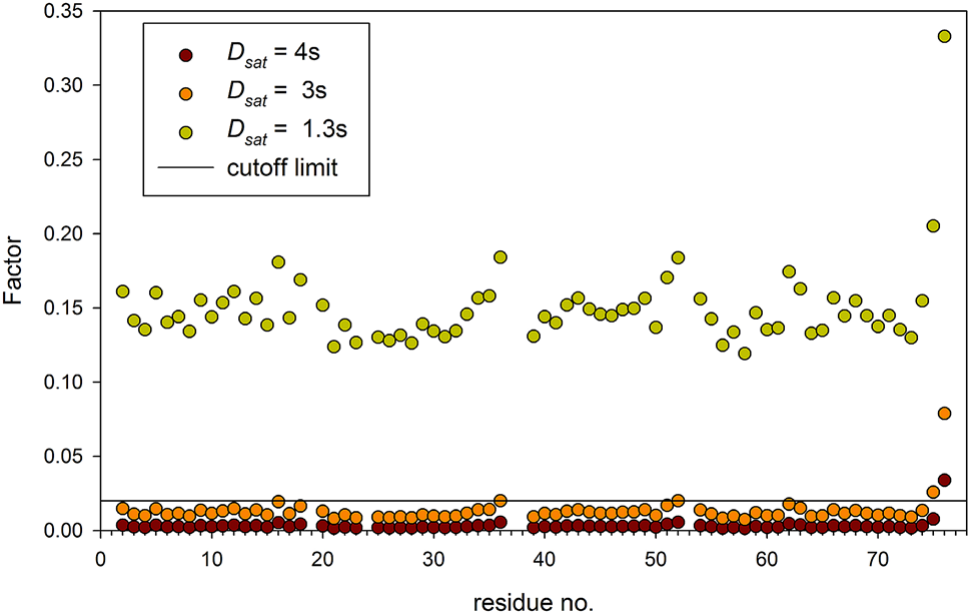
Factor characterizing efficiencies of the saturation of nitrogen magnetization for different *D*_*sat*_ values were calculated using residue specific *R*_1*N*_ values determined at 22.3 T in a separate measurement. A common sense but arbitrary limit 0.02 is marked with a horizontal line

**Fig. 8:**
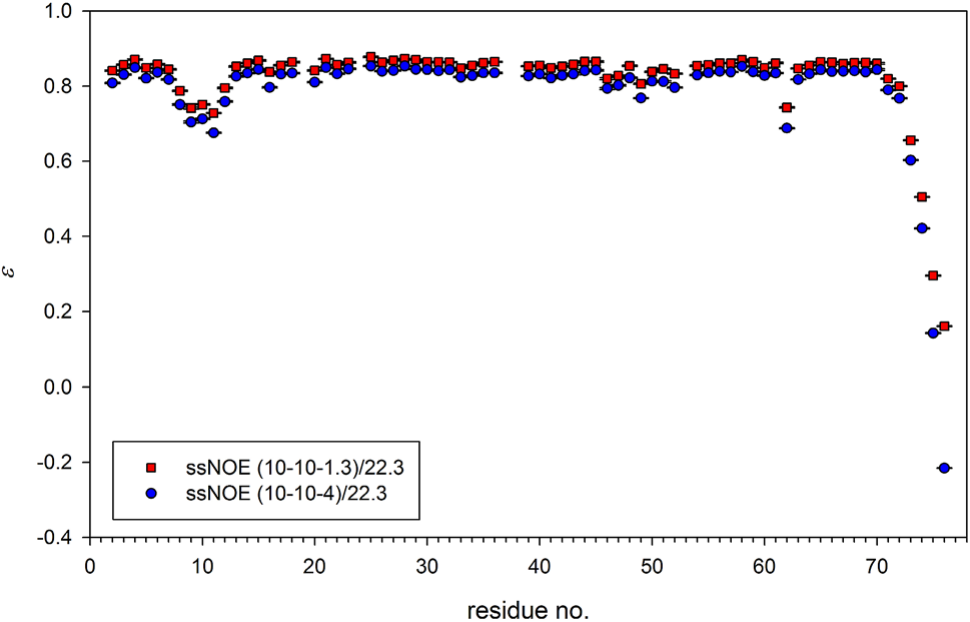
Nuclear Overhauser effect values obtained in steady-state NOE experiments with the saturation period *D*_*sat*_ set to 1.3 s (red squares) or 4 s (blue circles)

Calculation of factors $\exp ( - D_{sat} \cdot R_{1N} )$ using residue specific *R*_1*N*_ data is presented in Fig. 7 for *D*_*sat*_ values utilized in the measurements performed at 22.3 T as listed in Table S3. The *D*_*sat*_ = 3 s is sufficiently long for all residues except the last two C-terminal glycines, G75 and G76. In fact, even *D*_*sat*_ = 4 s is not long enough for the observation of unperturbed G76. Therefore, it is not surprising that *D*_*sat*_ = 1.3 s is much too short, and *ε*_*app*_ values derived from the experiment (10-10-1.3)/22.3 are significantly larger than those obtained at the longer period of *D*_*sat*_ = 4 s (Fig. 8), on average, 0.0348.

**Fig. 9:**
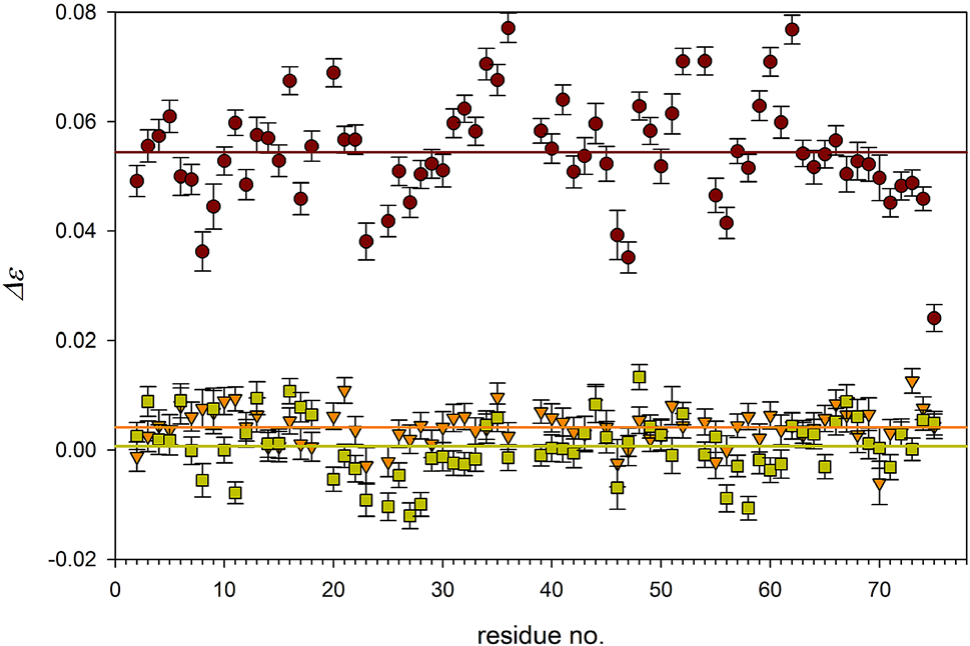
NOE differences Δ*ε* = *ε*_*app*_ − *ε* obtained for measurements performed at 22.3 T: ssNOE(13-0-3)/22.3, ssNOE(10-10-3)/22.3 (extracted from DNOE), ssNOE(6-0-3)/22.3, and ssNOE(3-0-3)/22.3. Δ for the *RD*_1_ pair: 3 s and 13 s (brown circles), the *RD*_1_ pair: 6 s and 13 s (orange triangles), the *RD*_1_ pair: 10 s and 13 s(light green squares). Color coded average differences after rejection of G76 with *ε* < 0 are equal to 0.0544, 0.0042, and 0.0007

The effect of a very short *RD*_1_ delay can be demonstrated by comparing experiments ssNOE(13-0-3)/22.3, ssNOE(10-10-3)/22.3, ssNOE(6-0-3)/22.3, and ssNOE(3-0-3)/22.3 (Fig. 9). The *RD*_1_ = 3 s and *RD*_1_ = 6 s result in the increase of *ε* magnitudes relative to the *RD*_1_ = 13 s on average, 0.0544 and 0.0042, respectively. On the other hand, average difference between measurements with *RD*_1_ = 13 s and *RD*_1_ = 10 s is negligible − 0.0007. This result gives evidence that *RD*_1_ delay equal to 10 s allows to reach the equilibrium state of H_N_ protons in the studies system.

Concluding, comparison of the NOE values obtained at different settings of *D*_*sat*_ or *RD*_1_ highlights the importance of the correct choice of delays in the determination of accurate ε values.

### Correction factors

As has been shown above, the effect of slow spin-lattice relaxation of water protons and the chemical exchange of amide protons with water combined with too short relaxation delays in the steady-state NOE experiments usually results in substantial systematic NOE errors owing to the incomplete relaxation towards the steady-state or equilibrium ^15^N polarization. Therefore, several correction factors were introduced to compensate such errors using the following equation

5 $$\varepsilon = \frac{{(1 - X)\varepsilon_{app} }}{{1 - X\varepsilon_{app} }}$$

where ε and ε_*app*_ are exact and apparent NOE values, respectively.

It has been claimed that the effect of incomplete *R*_1*W*_ recovery can be corrected by substituting the factor

5A $$X = \exp ( - RD \cdot R_{1W} )$$

into Eq. 5 (Skelton et al. 1993). It has been also suggested that factor

5B $$X = \exp ( - RD \cdot R_{1H} )$$

allows for the correction of the not sufficiently long relaxation delay *RD* with respect to *R*_1*H*_ (Grzesiek and Bax 1993). Another correction that takes into consideration the inconsistency of both *R*_1*N*_ and *R*_1*H*_ with relaxation delays has also been recommended (Freedberg et al. 2002):

5C $$X = \frac{{R_{1N} }}{{R_{1N} - R_{1H} }}\frac{{\exp ( - RD \cdot R_{1N} ) - \exp ( - RD \cdot R_{1H} )}}{{\exp ( - RD \cdot R_{1N} ) - 1}}$$

**Fig. 10:**
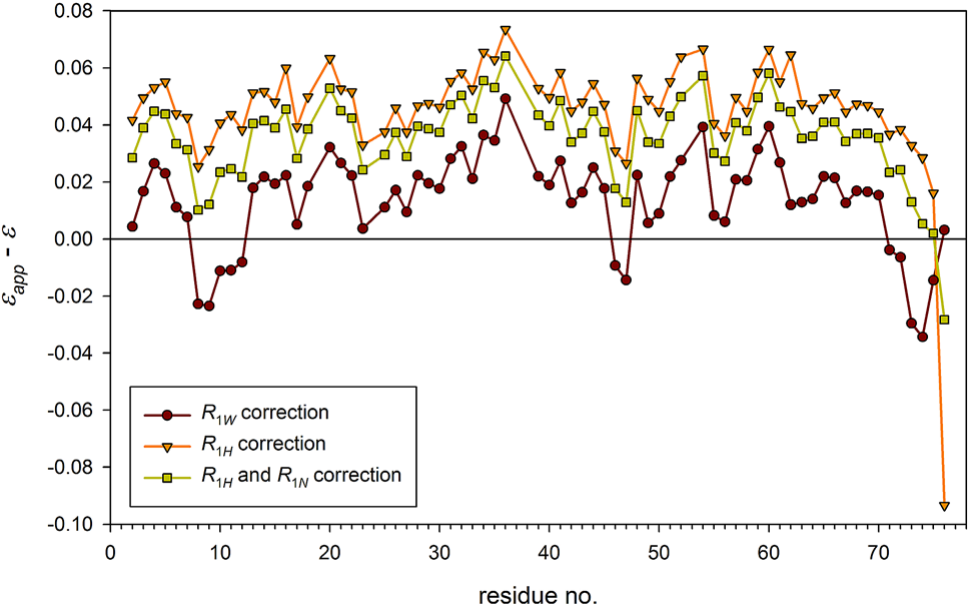
Residue specific differences between corrected *ε*_*app*_ and *ε* values obtained in (13-0-3)/22.3 measurement. The *ε*_*app*_ values were obtained from (3-0-3)/22.3 experiment after compensation for *R*_1*W*_ (Eq. 5A, brown circles), *R*_1*H*_ (Eq. 5B, orange triangles), and *R*_1*H*_, *R*_1*N*_ (Eq. 6, light green squares). Horizontal color-coded lines correspond to appropriate means of difference magnitudes

Efficiencies of all three corrections were checked on the NOE measurement with the intentionally too short delays: *RD*_1_ = 3 s, *RD*_2_ = 0, and *D*_*sat*_ = 3 s, (3-0-3)/22.3. As shown earlier (Fig. 9), all ε_*app*_ in (3-0-3)/22.3 measurement were larger than corresponding ε values in the correctly performed measurement (13-0-3)/22.3. The mean of differences was equal to 0.054. None of these above-listed corrections was able to fully compensate the effect of wrong adjustment of *RD*_1_ delay. Three corrections allowing for *R*_1*W*_ (Eq. 5), *R*_1*H*_ (Eq. 5B), and *R*_1*H*_ and *R*_1*N*_ (Eq. 5C) resulted in the means of absolute differences equal to 0.019, 0.048, and 0.036, respectively (Fig. 10). Therefore, these corrections have compensated for the delay missetting by 67%, 17%, and 38%, respectively*.* Obviously, the *R*_1*W*_ effect is the most important factor for compensation.

Compensation for a not long enough *D*_*sat*_ period with properly chosen *RD*_1_ is an easier task. The experiment (10-10-1.3)/22.3 was discussed earlier, and its results were shown in Fig. 8. Use of another correction,

6 $$\varepsilon = \frac{{\varepsilon_{app} - X}}{1 - X},{\text{where}},X = \exp ( - D_{sat} \cdot R_{1N} )$$

results in the corrected *ε*_*app*_ values, which differ from the DNOE experiment by an average of 0.003 (Fig. S7). Nevertheless, in view of the above-mentioned results, it is obvious that none of the existing correction terms should be used as a substitute for a properly designed experiment.

## Conclusions

In this study, it has been shown that dynamic NOE measurement is an efficient and accurate method for NOE determination. In particular, it presents its usefulness in cases of NOE values that are close to zero. This method provides a robust and more accurate alternative to widely used steady-state NOE measurement. The DNOE measurement allows for the determination of NOE values and their accuracies with standard nonlinear regression methods. If high accuracy longitudinal relaxation rates *R*_1_ are not of great importance, they can be simultaneously obtained with a reduced accuracy as a "by-product" in the DNOE data processing without any significant reduction of the accuracy and precision of determined NOE values.

It has been proven that commonly used methods of NOE accuracy based on the signal-to-noise ratio accompanying steady-state NOE measurements are reliable provided that root-mean-square noise has been determined correctly.

It has to be stressed that in view of the results presented in this work, none of the existing correction terms are able to restore accurate NOE values in cases where measurements are improperly set up and performed.

## Electronic supplementary material

Below is the link to the electronic supplementary material.

Electronic supplementary material 1 (PDF 1100 kb)
