# Supplementary material for: Dynamic 15N{1H} NOE measurements: a tool for studying protein dynamics
Source: J Biomol NMR. 2020 Sep 12;74(12):707–16. doi: 10.1007/s10858-020-00346-6 (PMC7701129; doi:10.1007/s10858-020-00346-6)

Supplementary material

Dynamic $^{15}N\{^{1}H\}$ NOE measurements – a tool for studying protein dynamics

Vladlena Kharchenko [1], Michal Nowakowski [1,2], Mariusz Jaremko [1], Andrzej Ejchart [3], Łukasz Jaremko [1*]

1. Division of Biological and Environmental Sciences and Engineering (BESE), King Abdullah University of Science and Technology (KAUST), Thuwal, 23955-6900, Saudi Arabia.
2. Faculty of Chemistry, Biological and Chemical Research Centre, University of Warsaw, Żwirki i Wigury 101, 02-089, Warszawa, Poland.
3. Institute of Biochemistry and Biophysics, Polish Academy of Sciences, Pawinskiego 5A, 02-106, Warszawa, Poland.

*e-mail: lukasz.jaremko@kaust.edu.sa

Table of Contents

Table S1	Minimal values of D_{sat}	p. 2
Figure S1	The dR_1 propagation in the processing of DNOE (16.4 T)	p. 2
Figure S2	The dR_1 propagation in the processing of DNOE (22.3 T)	p. 2
Figure S3	NOE errors, $d\varepsilon$, in different data processing of DNOE (16.4 T)	p. 3
Figure S4	The dR_1 errors in different data processing of DNOE (16.4 T)	p. 3
Figure S5	NOE errors, $d\varepsilon$, in different data processing of DNOE (22.3 T)	p. 4
Figure S6	The dR_1 errors in different data processing of DNOE (22.3 T)	p. 4
Table S2	Numerical results of different data processing of DNOE (22.3 T)	pp. 5-8
Determination of *signal-to-noise* ratio		p. 8
Table S3	Basic delays used in NOE and R_1 measurements	p. 9
Figure S7	Correction of the H_N saturation period D_{sat} (22.3 T)	p. 10

Table S1
The largest, R_1(D21), and the smallest, R_1(G76), ^{15}N longitudinal relaxation rates in ubiquitin. Minimal values of D_{sat} calculated from the condition $\exp(-D_{sat} \cdot R_{1N}) = 0.02$.

B_0 [T]	R_1(G76) [s^{-1}]	R_1(D21) [s^{-1}]	D_{sat}(G76) [s]	D_{sat}(D21) [s]
16.4	0.80	1.98	4.9	2.0
18.8	0.80	1.73	4.9	2.3
22.3	0.85	1.61	4.6	2.4

Figure S1
Visualization of the influence of dR_1 propagation in the sequential processing of R_1 and DNOE data at 16.4 T. Standard errors, $d\varepsilon$, neglecting inaccuracies in R_1 determination (yellow squares) or accounting for dR_1 propagation (blue triangles). The differences are visible for G75 ($\varepsilon = -0.248$) and G76 ($\varepsilon = -0.812$).

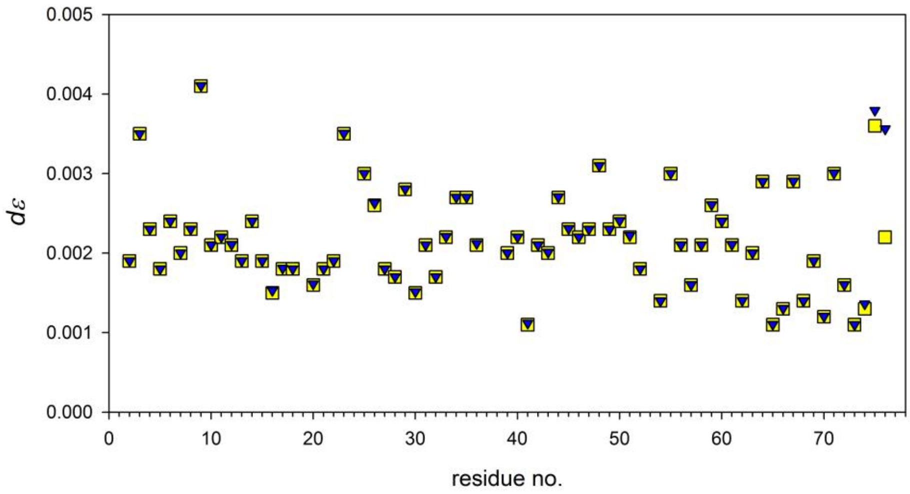

Figure S2
Visualization of the influence of dR_1 propagation in the sequential processing of R_1 and DNOE data at 22.3 T. Standard errors, $d\varepsilon$, neglecting inaccuracies in R_1 determination (yellow squares) or accounting for dR_1 propagation (blue triangles). The differences are visible for G75 ($\varepsilon = 0.142$) and G76 ($\varepsilon = -0.246$).

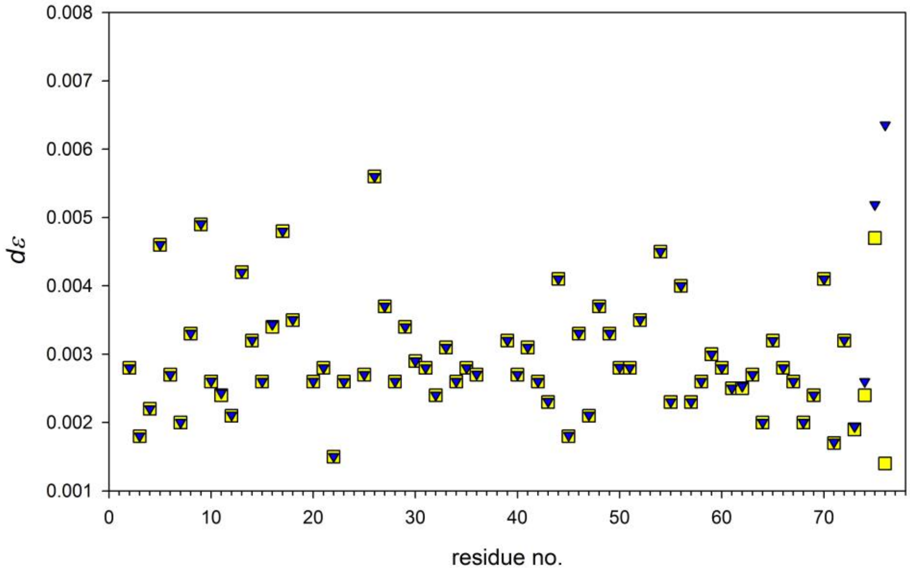

Figure S3

Comparison of NOE errors, $d\varepsilon$, determined by different methods of data processing of DNOE data measured at 16.4 T: sequential task with dR_1 propagation (brown circles), simultaneous task (orange triangles), stand-alone DNOE data (light green squares).

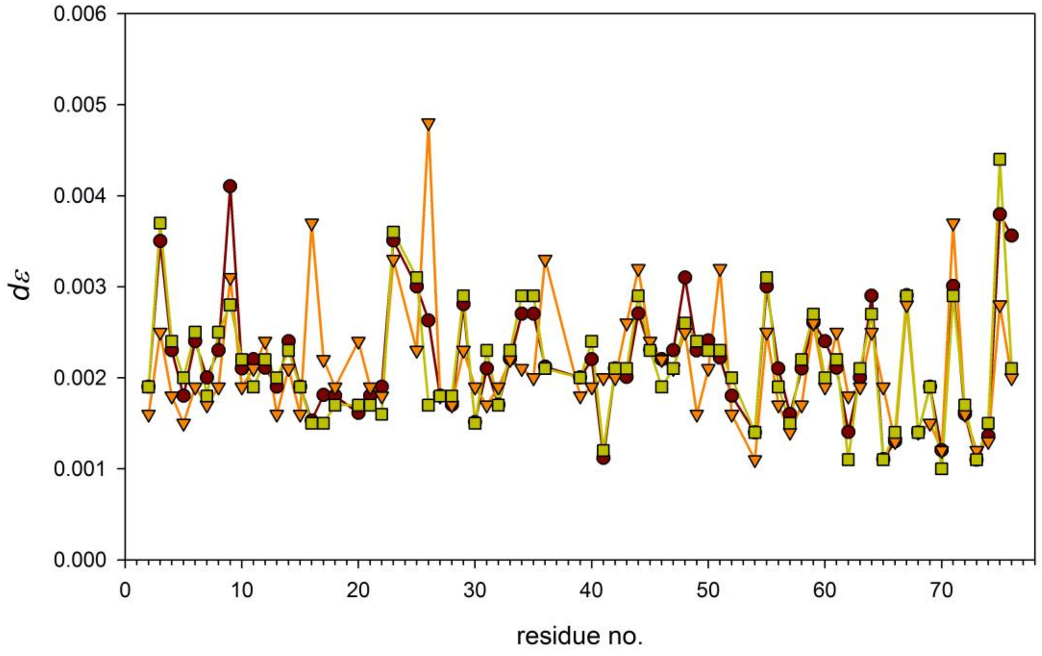

Figure S4

Comparison of R_1 errors, dR_1, determined in different methods of data processing of DNOE data measured at 16.4 T: independent R_1 measurement delivering results to the sequential task (brown circles), simultaneous task (orange triangles), stand-alone DNOE data (light green squares).

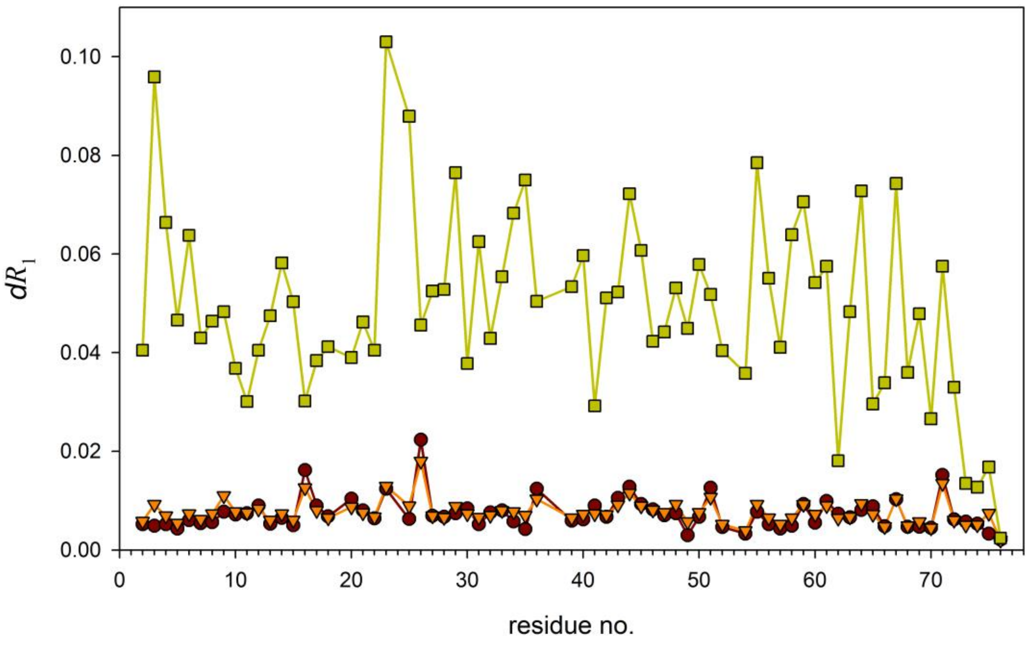

Figure S5

Comparison of NOE errors, $d\varepsilon$, determined by different methods of data processing of DNOE data measured at 22.3 T: sequential task with dR_1 propagation (brown circles), simultaneous task (orange triangles), stand-alone DNOE data (light green squares).

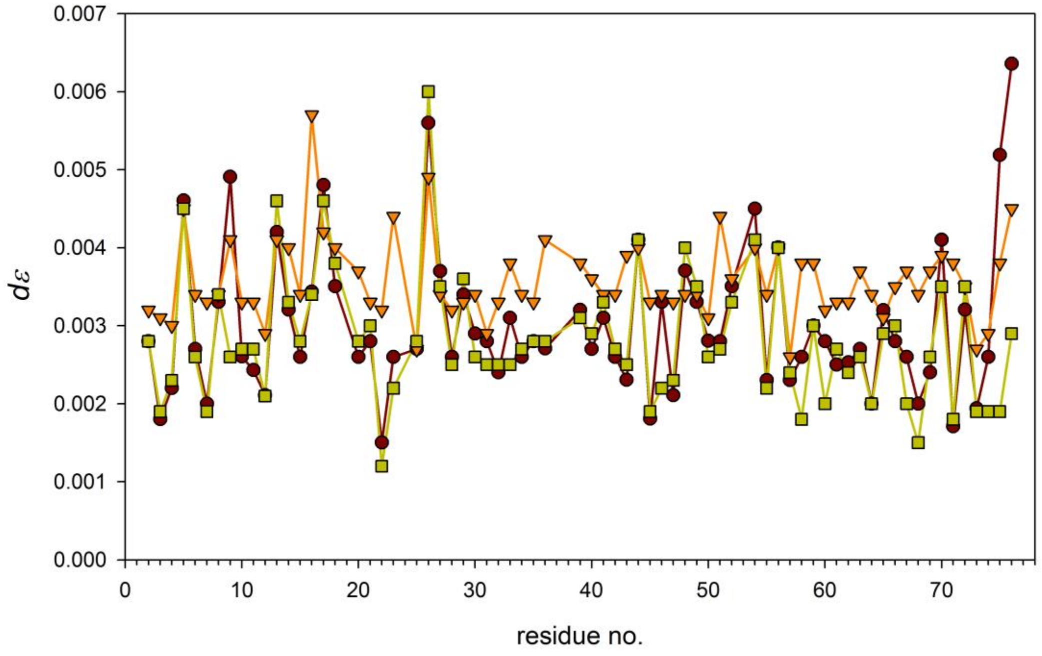

Figure S6

Comparison of R_1 errors, dR_1, determined in different methods of data processing of DNOE data measured at 22.3 T: independent R_1 measurement delivering results to the sequential task (brown circles), simultaneous task (orange triangles), stand-alone DNOE data (light green squares).

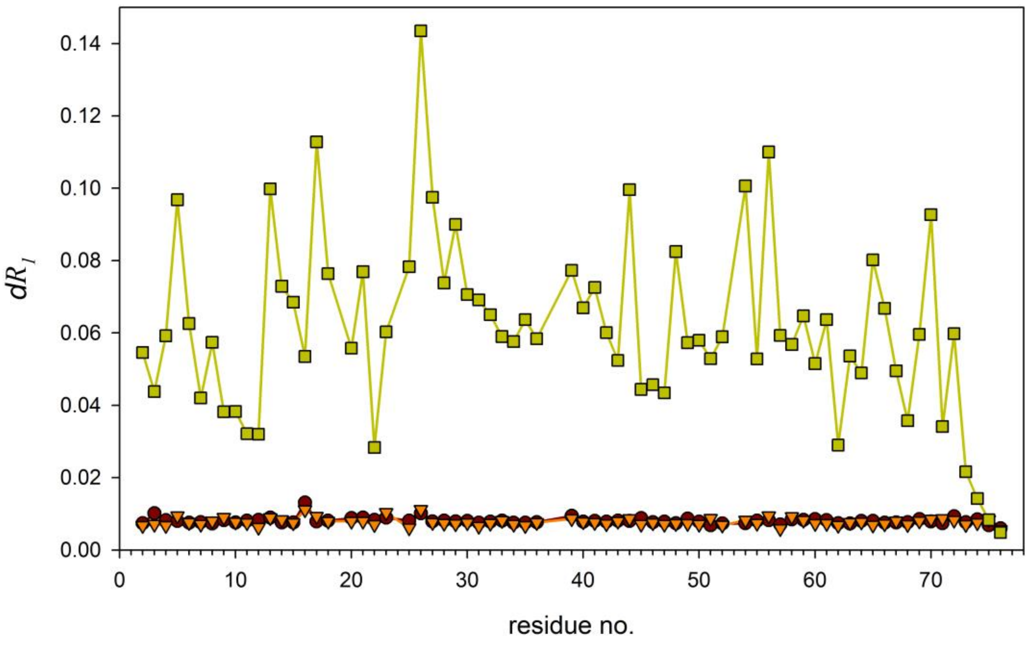

Table S2

Results of three different data processing of dynamic NOE at 22.3 T: sequential determination of R_1 from the R_1 dedicated data followed by the ε determination from DNOE measurement utilizing previously determined R_1 values, simultaneous use of DNOE and R_1 data in a single computational task, and stand-alone DNOE data used for the determination of both, R_1 and ε values.

Part A: ε values and their standard deviations; sequential $d\varepsilon$ account for dR_1 propagation

ε	Seq. R_1 and DNOE		Sim. R_1 and DNOE		Stand-alone DNOE data	
Q2	0.807	0.003	0.807	0.003	0.808	0.003
I3	0.831	0.002	0.831	0.003	0.831	0.002
F4	0.848	0.002	0.848	0.003	0.848	0.002
V5	0.818	0.005	0.818	0.005	0.819	0.005
K6	0.834	0.003	0.834	0.003	0.834	0.003
T7	0.817	0.002	0.817	0.003	0.818	0.002
L8	0.748	0.003	0.748	0.003	0.749	0.003
T9	0.701	0.005	0.701	0.004	0.704	0.003
G10	0.712	0.003	0.713	0.003	0.713	0.003
K11	0.677	0.002	0.677	0.003	0.677	0.003
T12	0.756	0.002	0.756	0.003	0.756	0.002
I13	0.825	0.004	0.825	0.004	0.825	0.005
T14	0.829	0.003	0.830	0.004	0.830	0.003
L15	0.840	0.003	0.840	0.003	0.840	0.003
E16	0.799	0.003	0.799	0.006	0.797	0.003
V17	0.828	0.005	0.828	0.004	0.829	0.005
E18	0.831	0.004	0.831	0.004	0.831	0.004
S20	0.810	0.003	0.810	0.004	0.811	0.003
D21	0.848	0.003	0.848	0.003	0.847	0.003
T22	0.833	0.002	0.833	0.003	0.833	0.001
I23	0.844	0.003	0.844	0.004	0.844	0.002
N25	0.854	0.003	0.854	0.003	0.854	0.003
V26	0.843	0.006	0.843	0.005	0.843	0.006
K27	0.844	0.004	0.844	0.003	0.844	0.004
A28	0.854	0.003	0.854	0.003	0.855	0.003
K29	0.841	0.003	0.841	0.003	0.842	0.004
I30	0.844	0.003	0.844	0.003	0.845	0.003
Q31	0.843	0.003	0.843	0.003	0.844	0.003
D32	0.844	0.002	0.844	0.003	0.844	0.003
K33	0.823	0.003	0.823	0.004	0.824	0.003
E34	0.825	0.003	0.825	0.003	0.825	0.003
G35	0.834	0.003	0.834	0.003	0.835	0.003
I36	0.836	0.003	0.836	0.004	0.836	0.003
D39	0.829	0.003	0.829	0.004	0.830	0.003
Q40	0.832	0.003	0.832	0.004	0.832	0.003
Q41	0.820	0.003	0.820	0.003	0.821	0.003
R42	0.830	0.003	0.830	0.003	0.830	0.003
L43	0.830	0.002	0.830	0.004	0.830	0.003
I44	0.835	0.004	0.835	0.004	0.836	0.004
F45	0.840	0.002	0.840	0.003	0.840	0.002

A46	0.792	0.003	0.792	0.003	0.794	0.002
G47	0.801	0.002	0.801	0.003	0.801	0.002
K48	0.821	0.004	0.821	0.003	0.821	0.004
Q49	0.768	0.003	0.768	0.003	0.769	0.004
L50	0.813	0.003	0.813	0.003	0.813	0.003
E51	0.814	0.003	0.814	0.004	0.814	0.003
D52	0.798	0.004	0.798	0.004	0.799	0.003
R54	0.833	0.005	0.833	0.004	0.834	0.004
T55	0.831	0.002	0.831	0.003	0.832	0.002
L56	0.841	0.004	0.841	0.004	0.842	0.004
S57	0.838	0.002	0.838	0.003	0.838	0.002
D58	0.853	0.003	0.853	0.004	0.853	0.002
Y59	0.837	0.003	0.837	0.004	0.836	0.003
N60	0.828	0.003	0.828	0.003	0.829	0.002
I61	0.836	0.003	0.836	0.003	0.836	0.003
Q62	0.687	0.003	0.687	0.003	0.688	0.002
K63	0.816	0.003	0.816	0.004	0.816	0.003
E64	0.833	0.002	0.833	0.003	0.833	0.002
S65	0.847	0.003	0.847	0.003	0.847	0.003
T66	0.838	0.003	0.838	0.004	0.838	0.003
L67	0.836	0.003	0.836	0.004	0.837	0.002
H68	0.838	0.002	0.838	0.003	0.839	0.002
L69	0.835	0.002	0.835	0.004	0.835	0.003
V70	0.837	0.004	0.837	0.004	0.838	0.004
L71	0.791	0.002	0.791	0.004	0.791	0.002
R72	0.767	0.003	0.767	0.004	0.766	0.004
L73	0.601	0.002	0.602	0.003	0.602	0.002
R74	0.417	0.003	0.417	0.003	0.420	0.002
G75	0.127	0.005	0.127	0.004	0.139	0.002
G76	-0.257	0.006	-0.259	0.005	-0.258	0.003

Part B: R_1 values and their standard deviations

$\boldsymbol{R_1}$ [s^{-1}]	Seq. R_1 and DNOE		Sim. R_1 and DNOE		Stand-alone DNOE data	
Q2	1.405	0.007	1.408	0.007	1.462	0.055
I3	1.504	0.010	1.534	0.007	1.540	0.044
F4	1.538	0.008	1.537	0.007	1.568	0.059
V5	1.409	0.008	1.420	0.009	1.533	0.097
K6	1.511	0.008	1.514	0.008	1.593	0.063
T7	1.490	0.008	1.485	0.007	1.553	0.042
L8	1.545	0.007	1.546	0.008	1.600	0.057
T9	1.433	0.008	1.427	0.009	1.614	0.038
G10	1.491	0.008	1.503	0.008	1.525	0.038
K11	1.442	0.008	1.450	0.008	1.427	0.032
T12	1.404	0.008	1.401	0.006	1.447	0.032
I13	1.498	0.009	1.497	0.009	1.505	0.100
T14	1.427	0.008	1.433	0.008	1.476	0.073
L15	1.521	0.008	1.525	0.008	1.527	0.069
E16	1.316	0.013	1.314	0.011	1.241	0.054

V17	1.495	0.008	1.497	0.009	1.650	0.113
E18	1.368	0.008	1.367	0.008	1.341	0.076
S20	1.450	0.009	1.454	0.008	1.473	0.056
D21	1.607	0.009	1.614	0.008	1.560	0.077
T22	1.521	0.008	1.515	0.007	1.593	0.028
I23	1.589	0.009	1.611	0.010	1.719	0.060
N25	1.568	0.008	1.557	0.006	1.621	0.078
V26	1.581	0.010	1.580	0.011	1.502	0.144
K27	1.560	0.008	1.559	0.008	1.694	0.098
A28	1.592	0.008	1.594	0.007	1.685	0.074
K29	1.517	0.008	1.516	0.007	1.582	0.090
I30	1.544	0.008	1.546	0.008	1.674	0.071
Q31	1.565	0.008	1.567	0.007	1.685	0.069
D32	1.543	0.008	1.542	0.007	1.575	0.065
K33	1.482	0.008	1.477	0.008	1.621	0.059
E34	1.426	0.008	1.430	0.007	1.474	0.058
G35	1.419	0.008	1.419	0.007	1.486	0.064
I36	1.302	0.008	1.301	0.008	1.352	0.058
D39	1.564	0.009	1.556	0.009	1.659	0.077
Q40	1.490	0.008	1.485	0.008	1.523	0.067
Q41	1.513	0.008	1.512	0.008	1.565	0.073
R42	1.449	0.008	1.452	0.007	1.512	0.060
L43	1.427	0.008	1.417	0.008	1.417	0.052
I44	1.464	0.008	1.467	0.009	1.560	0.100
F45	1.481	0.009	1.480	0.007	1.492	0.044
A46	1.487	0.008	1.488	0.008	1.638	0.046
G47	1.465	0.008	1.464	0.007	1.488	0.043
K48	1.461	0.007	1.463	0.007	1.450	0.083
Q49	1.427	0.009	1.424	0.007	1.447	0.057
L50	1.530	0.008	1.532	0.007	1.618	0.058
E51	1.361	0.007	1.363	0.009	1.442	0.053
D52	1.304	0.007	1.302	0.007	1.399	0.059
R54	1.429	0.007	1.430	0.008	1.596	0.101
T55	1.498	0.008	1.495	0.007	1.570	0.053
L56	1.601	0.008	1.608	0.009	1.718	0.110
S57	1.548	0.007	1.551	0.006	1.608	0.059
D58	1.636	0.008	1.656	0.009	1.812	0.057
Y59	1.476	0.008	1.479	0.008	1.391	0.065
N60	1.539	0.009	1.538	0.007	1.691	0.052
I61	1.532	0.008	1.531	0.007	1.524	0.064
Q62	1.344	0.007	1.340	0.007	1.388	0.029
K63	1.396	0.007	1.403	0.008	1.472	0.054
E64	1.552	0.008	1.553	0.008	1.602	0.049
S65	1.541	0.008	1.537	0.007	1.670	0.080
T66	1.426	0.008	1.432	0.007	1.424	0.067
L67	1.488	0.008	1.487	0.008	1.618	0.050
H68	1.435	0.008	1.434	0.007	1.532	0.036
L69	1.487	0.009	1.494	0.008	1.502	0.060

V70	1.526	0.008	1.523	0.009	1.714	0.093
L71	1.485	0.007	1.524	0.009	1.502	0.034
R72	1.538	0.009	1.530	0.008	1.521	0.060
L73	1.570	0.008	1.575	0.007	1.602	0.022
R74	1.435	0.008	1.438	0.008	1.482	0.014
G75	1.219	0.007	1.216	0.008	1.295	0.008
G76	0.846	0.006	0.843	0.006	0.846	0.005

Determination of *signal-to-noise* ratio

All processed NMR spectra were analyzed with SPARKY software. The '*lt*' command delivers list of the selected cross-peaks containing, besides other data, peak intensities and their signal-to noise ratios (*SNR*) often indispensable in quantitative studies. On the other hand, SPARKY program contains an option to provide root-mean-square noise (*RMSN*) calculated for manually selected region of a 2D spectrum applying '*rm*' command. The *SNR* values calculated using the *RMSN* data differ from those delivered by the '*lt*' command. The ratios *SNR*(*lt*)/*SNR*(*rm*) determined in measurements used in this work (71 values with an average equal to 1.26) are visualized with a histogram shown below. Generally, *SNR*(*lt*) are greater than *SNR*(*rm*) resulting in the underestimation of NOE errors, $d\varepsilon$. Hence, the use of NOE data in the protein dynamics evaluation becomes unreliable. The use of *SNR*(*rm*) option is strongly recommended in all studies requiring quantitative information.

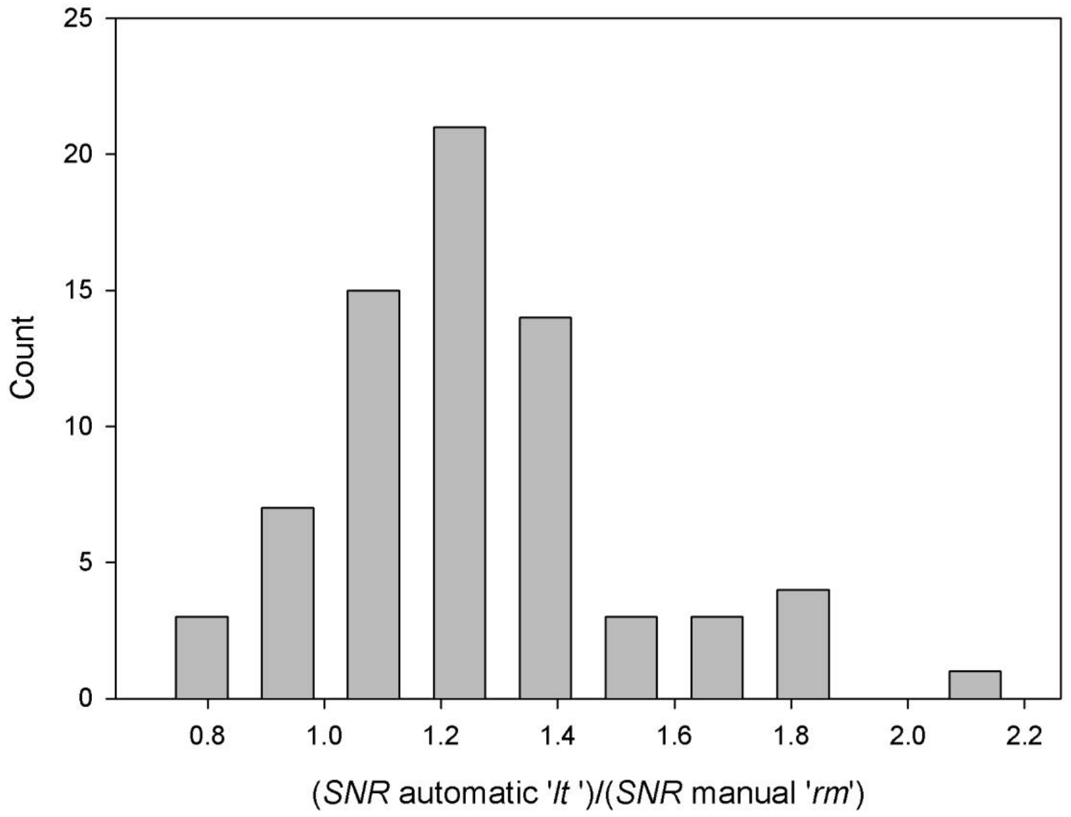

Table S3
General data for NOE and R_1 measurements presented and analyzed in the text.

Exp	B_0 [T]	RD_1 [s]	RD_2 [s]	D_{sat} [s]	T_{exp} [h] [g]	<*SNR*> [h]	$\exp(-RD_i \cdot R_{1W})$
DNOE	16.4	10.0	10.0	set [c]	74.0/19.5	405	0.020
DNOE	22.3	10.0	10.0	set [d]	54.5/19.7	791	0.016
ssNOE	18.8	14.0	0.0	4.0	8.1	569	0.003
ssNOE	18.8	14.0	0.0	7.0	9.4	617	0.003
ssNOE	18.8	14.0	0.0	14.0	12.5	610	0.003
ssNOE	22.3	13.0	0.0	3.0	7.2	681	0.005
ssNOE [a]	22.3	10.0	0.0	10.0	9.0	624	0.016
ssNOE [b]	16.4	10.0	10.0	8.0	12.5	405	0.020
ssNOE [b]	16.4	10.0	10.0	6.01	11.6	405	0.020
ssNOE [b]	16.4	10.0	10.0	4.01	10.8	405	0.020
ssNOE [b]	22.3	10.0	10.0	4.01	10.6	791	0.016
ssNOE [b]	22.3	10.0	10.0	3.0	10.3	791	0.016
ssNOE [b]	22.3	10.0	10.0	1.3	9.6	791	0.016
ssNOE	22.3	6.0	0.0	3.0	4.1	682	0.084
ssNOE	22.3	3.0	0.0	3.0	2.8	638	0.290
R_1	16.4	4.0	--	set [e]			--
R_1	22.3	4.0	--	set [f]			--

[a] series of 10 separate measurements performed with identical spectrometer setup
[b] ssNOE was determined using two spectra taken from DNOE measurement with D_{sat}=0 for the reference measurement and D_{sat} value given in column 5.
[c] set of thirteen D_{sat} values [0.00, 0.09, 0.18, 0.31, 0.44, 0.66, 1.06, 2.00, 2.51, 4.01, 6.01, 8.00, 10.00].
[d] set of eleven D_{sat} values [0.00, 0.11, 0.22, 0.35, 0.55, 0.66, 0.79, 1.10, 1.30, 3.00, 4.01].
[e] set of seventeen R_1 evolution times [0.03, 0.03, 0.05, 0.08, 0.11, 0.15, 0.19, 0.25, 0.33, 0.44, 0.58, 0.77, 1.04, 1.45, 1.45, 1.90, 1.90].
[f] set of seventeen R_1 evolution times [0.01, 0.01, 0.02, 0.03, 0.07, 0.10, 0.14, 0.19, 0.29, 0.33, 0.40, 0.54, 0.71, 0.94, 1.27, 1.50, 1.80].
[g] total experimental time reported in hours. For DNOE experiments first number corresponds to full measurement with redundant delays. The second number gives experimental time for the curtailed DNOE experiment composed of four D_{sat} values (see section "Setup and data processing of DNOE measurement" and Fig. 3).
[h] *SNR* values averaged over 66 residues (Q2 - R72) determined for non-saturated (D_{sat}=0) run.

Figure S7

Correction of the H_N saturation period D_{sat}. Apparent NOE values, ε_{app}, determined in the experiment (10-10-1.3)/22.3 are noticeably larger than those in one of the most accurate measurement DNOE/22.3. Their differences $\Delta\varepsilon = \varepsilon_{app} - \varepsilon$ are shown as red squares. Differences for three C-terminal residues are out of scale (0.088, 0.168, 0.419). Use of the correction described in the section Correction factors (Eq. 7), significantly diminished the difference (blue circles), on average from 0.037 (dashed red line) to 0.003 (dashed blue line).

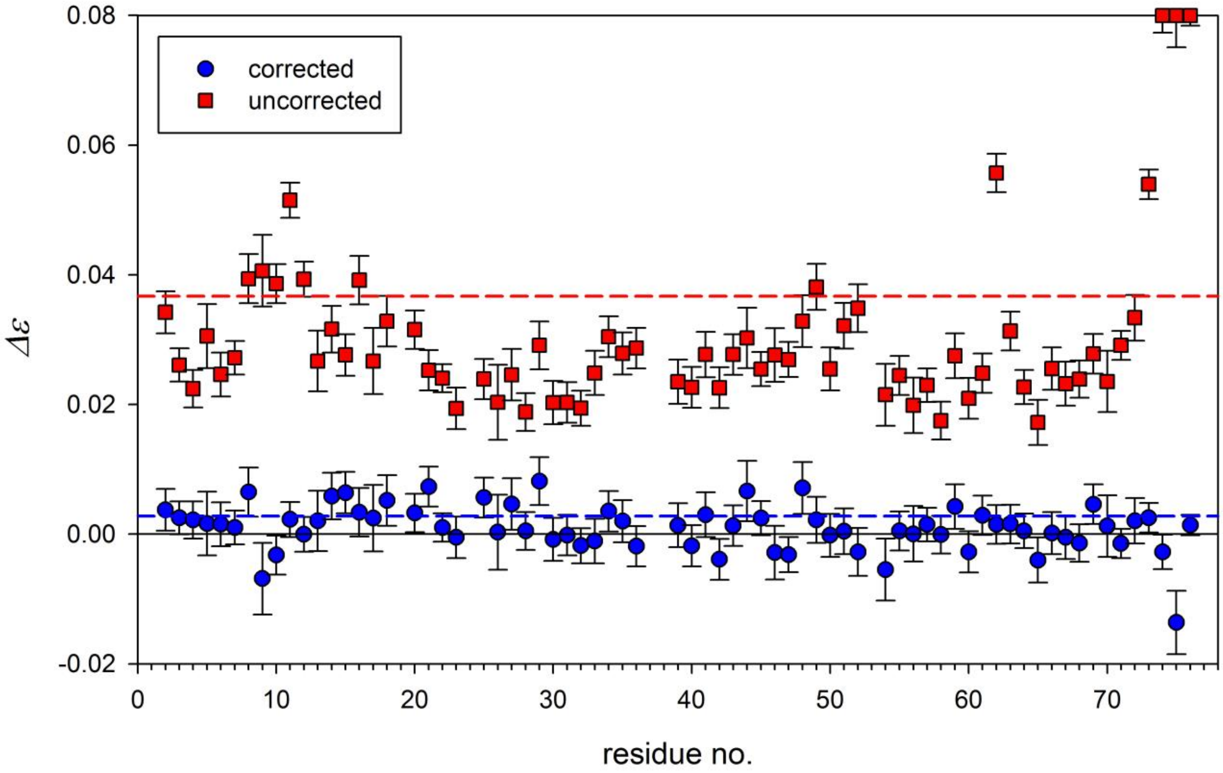

Supplement: Supplementary file 1 — Electronic supplementary material 1 (PDF 1100 kb) [file 10858_2020_346_MOESM1_ESM.pdf]
